# Profiles of digital empowerment among older adults and their association with self-perception of aging: a latent profile analysis

**DOI:** 10.1186/s12877-026-08055-9

**Published:** 2026-07-31

**Authors:** Lei Zhang, Longtao Wang, Weichao Chen

**Affiliations:** School of Journalism and Communication, Hunan Normal University, Changsha, 41000 China

**Keywords:** Digital empowerment, Self-perception of aging, Internet use skills, Older adults, Latent profile analysis

## Abstract

**Objective:**

The relationship between digital empowerment patterns and self-perception of aging (SPA) among older adults remains complex and understudied. This research investigates how digital empowerment patterns were associated with SPA, with particular attention to the moderating role of Internet use skills.

**Methods:**

Data were collected from the 2020 China Longitudinal Aging Social Survey (CLASS), including 3,032 participants aged 60 and above. Latent Profile Analysis (LPA) was employed to systematically identify distinct digital empowerment patterns.

**Results:**

The research identified four distinct digital empowerment patterns: Class 1 (Social digital empowerment pattern, 18.6%), Class 2 (High digital empowerment pattern, 19.8%), Class 3 (Entertainment digital empowerment pattern, 22.3%), and Class 4 (Low digital empowerment pattern, 39.3%). Compared to the Class 4, all other patterns were significantly associated with higher SPA scores, with the Class 3 demonstrating the strongest positive association. Internet use skills significantly moderated the associations for specific digital empowerment profiles, particularly the high and entertainment-oriented profiles. Gender differences revealed that males benefited more from the Class 1 and Class 2, while females experienced stronger positive effects from the Class 3. Rural older adults and those with lower educational attainment showed consistently stronger associations between digital empowerment and SPA.

**Conclusion:**

This study demonstrates that digital engagement is significantly and positively associated with SPA among older adults, with Internet use skills serving as an important moderating factor. Three interventions are needed: age-friendly interfaces, targeted engagement strategies, and digital literacy programs. Bridging the digital divide is essential for positive aging.

## Introduction

Self-perception of aging (SPA) referred to individuals' view of themselves becoming older [1], specifically encompassing a range of age stereotypes and beliefs relevant to specific life domains [2, 3]. SPA play a pivotal role in the life quality and overall well-being of older adults [4]. Positive perceptions of aging are linked to better cognitive functioning and healthier behaviors, whereas negative perceptions have been associated with higher risks of depression and poorer health outcomes [5, 6].

With the increasing accessibility of the Internet, older adults’ engagement with digital technologies has grown substantially [7]. Amichai-Hamburger and Ben-Artzi [8] noticed that the Internet has evolved from a relatively simple and unified platform into a more complex network containing various services and functions. Schehl et al. [9] has identified three primary motivations underlying older adults’ online activities: accessing information, maintaining social connections, and completing daily tasks. Digital empowerment refers to older adults’ perceived benefits and enhanced capabilities gained through Internet use [10]. It encompassing five dimensions: economic, political, security, public services, and self-actualization [11]. Compared with younger populations, older adults tend to show lower levels of engagement in digital political, professional, and economic activities. Consequently, high-frequency daily life scenarios—such as transportation, healthcare, consumption, entertainment, and social interaction—were selected to assess digital empowerment among the older adults [12].

Digital services (such as online banking, video calling, gaming, and e-shopping) have become increasingly integrated into older adults’ daily lives and may contribute to active aging [13]. Köttl et al. [14] further reported that online shopping, e-banking, and video calling were perceived as being associated with youth and a modern lifestyle. Similarly, a qualitative study by Juárez et al. [15] showed that technology use helped older adults develop a sense of being younger and more connected to contemporary society, potentially reshaping their negative perceptions of technology and aging. Evidence from older adults in the United States suggests that computer use is positively associated with SPA, both directly and indirectly through enhanced self-efficacy [16]. In China, Wang [17] also found that Internet use was positively related to the attitudes toward aging among older adults. However, other studies have suggested that SPA may influence Internet use rather than result from it [18]. These mixed findings underscore the need to further clarify the mechanisms through which digital engagement is associated with older adults’ self-perceptions of aging.

Stereotype Embodiment Theory [19] provides a theoretical framework for understanding this relationship. The theory proposes that age-related stereotypes are gradually internalized from the social environment across the life course and become personally relevant when individuals perceive themselves as older adults. These internalized stereotypes may operate implicitly and influence how older adults evaluate their abilities, social roles, and adaptability, thereby contributing to more positive or negative self-perceptions of aging. From this perspective, heterogeneous digital empowerment profiles may have differential implications for SPA, as digital experiences are always embedded within broader social meanings attached to aging. Older adults frequently encounter stereotypes that portray them as incapable of learning new technologies, which may constrain their ability to translate digital engagement into meaningful benefits. Within this framework, internet use skills may serve as an important moderator that shapes how older adults interpret and benefit from their digital experiences. Those with stronger skills are better positioned to convert digital engagement into tangible opportunities for autonomy, social connection, and active participation, thereby amplifying the positive association between digital empowerment profiles and SPA. This theoretical reasoning thus justifies the examination of internet use skills as a potential moderator in the relationship between digital empowerment profiles and SPA.

Beyond clarifying the mechanism linking digital engagement to SPA, a comprehensive examination of this association also requires methodological attention to how digital engagement itself is conceptualized and measured. To the best of our knowledge, there are several studies investigating the association between Internet use and SPA outcomes. However, existing research adopted variable-centered approaches, which examine relationships among variables averaged across individuals and implicitly assume population homogeneity [20, 21]. Because digital engagement is a multidimensional concept, a person-centered method such as latent profile analysis (LPA) would be beneficial for identifying combinations of digital engagement indicators that jointly explain older adults' digital behavioral processes. "Latent profiles" identified from a person-centered approach are inferred by a variety of dimensions of observed indicators and can differ along these indicators quantitatively (level) and qualitatively (shape), which means that one profile could be high in different indicators, whereas another could be low in these indicators, and there could also be differences between profiles in different indicators [22].Compared to other classification models, LPA is a model-based approach that uses posterior probabilities estimated via maximum likelihood, with model fit indices and statistical diagnostic tools ensuring classification accuracy [23]. Using the LPA approach, we can identify the profiles of individuals with similar characteristics of digital engagement and thus capture the heterogeneity of different potential engagement profiles among older adults [24]. Because of these advantages, the LPA approach has been widely applied in subgroup classification research in fields such as sociology and psychology [25].

Using these analyses, this study aimed to answer the following two questions: (1) identify latent profiles of digital empowerment across multiple dimensions, and (2) examine the associations between these digital empowerment profiles and SPA among older adults.

## Methods

### Data and sample

We conducted a cross-sectional analysis with data from the China Longitudinal Aging Social Survey (CLASS) 2020, a nationally representative survey conducted by the National Survey Research Center at Renmin University of China. Launched in 2010 and conducted biennially, CLASS examines various aspects affecting older adults, including health, social relationships, and digital technology use. CLASS utilized a three-stage, probability-proportionate-to-size sampling technique. In the first stage, 134 counties/districts were selected from a comprehensive list of county-level units. In the second stage, 462 villages and communities were randomly selected, maintaining an urban-to-rural ratio of 6:4. Data collection involved face-to-face interviews using standardized questionnaires.

In this study, we used data from CLASS 2020, which included 11,398 participants. After excluding incomplete or invalid responses, 3,032 participants aged 60 and older were available for the analysis.

### Measurements

#### Dependent variable

Self-perception of aging was assessed by the Attitudes to Aging Questionnaire (AAQ) [26]. The AAQ was originally developed in English as a 24-item multidimensional measure of aging attitudes. The Chinese version of the AAQ was adapted and validated by Huang et al. [27], with psychometric properties including internal consistency and construct validity confirmed in Chinese community-dwelling older adults. Subsequent work by Gao et al. [28] further developed and psychometrically evaluated a brief Chinese version of the AAQ (AAQ-BC) specifically for use in large-scale surveys of older Chinese adults, confirming its three-factor structure, acceptable internal consistency, test–retest reliability, convergent validity, and discriminant validity.

Consistent with prior research [29], this validated scale includes 7 items: (a) I feel that I am already old; (b) In my opinion, aging is a process of continuous loss (e.g., loss of health, loss of friends and relatives, loss of ability, etc.); (c) I find it more difficult to make new friends as I age; (d) I feel that I am excluded because of my age; (e) The older you get, the better you are at dealing with life's problems; (f) Wisdom grows with age; (g) There are many pleasurable things about growing old.

Each item was rated on a five-point Likert scale ranging from 1 ("totally disagree") to 5 ("totally agree"). Four negatively worded items (items 1–4) were reverse-coded before calculating the total score. The SPA score was obtained by summing all seven items after reverse coding, with a theoretical range from 7 to 35. Higher scores indicate a more positive self-perception of aging. This scoring approach is consistent with previous studies using the same CLASS-embedded AAQ items [30], supporting the cross-study comparability of the measure.

The AAQ has demonstrated satisfactory psychometric properties in previous studies [31], including acceptable internal consistency and construct validity. In the current study, the scale showed acceptable reliability, with a Cronbach’s alpha coefficient of 0.701.

#### Independent variable

Consistent with prior research [32, 33], digital empowerment was measured by assessing the extent to which Internet technologies facilitate respondents’ daily activities across eight domains: social interaction, shopping and consumption, access to news, leisure and entertainment, travel and tourism, health services, investment and financial management, and learning and training.

Each item was rated on a three-point scale ranging from 1 (“inconvenient”), 2 (“no impact”), to 3 (“convenient”), with higher scores indicating a higher level of perceived digital empowerment.

The digital empowerment scale demonstrated good internal consistency reliability, with Cronbach’s α = 0.82. Construct validity was supported by exploratory factor analysis. The Kaiser–Meyer–Olkin value was 0.83, and Bartlett’s test of sphericity was significant (χ^2^ = 7198.835, df = 28, *p* < 0.001), indicating suitability for factor analysis. Exploratory factor analysis suggested a two-factor structure with factor loadings ranging from 0.316 to 0.823, explaining 40.2% of the total variance, supporting the construct validity of the digital empowerment measure in the present sample.

#### Moderating variable

The moderating variable in this study is Internet use skills. Respondents were asked to evaluate their proficiency in using three types of digital devices: (a) mobile phones, (b) desktop or laptop computers, and (c) tablets (e.g., iPad or similar devices). Each item was rated on a five-point response scale, with 1 indicating “very unskilled,” 2 indicating “unskilled,” 3 indicating “neutral,” 4 indicating “skilled,” and 5 indicating “very skilled.” Higher scores reflected greater digital proficiency among older adults. The Cronbach’s alpha coefficient for this scale was 0.801.

#### Covariates

We selected the following variables as sociodemographic characteristics of the study population. Age, gender, marital status, residence status(rural/urban), educational level, family annual income (logarithmic scale), employment status, living arrangement, activities of daily living (ADLs), instrumental activities of daily living (IADLs), number of comorbid chronic diseases, cognition, social participation, self-efficacy, and living region (Central/East/West). The measurement of key variables is described below.

Activities of Daily Living (ADLs) were assessed using 11 items (B4-1 to B4-11) drawn from the CLASS questionnaire, covering basic self-care tasks including telephoning, grooming, dressing, bathing, eating, medication management, urinary continence, bowel continence, toileting, transferring from bed to chair, and indoor mobility. Each item was scored on a three-point scale (0 = completely unable to perform, 1 = some assistance needed, 2 = no assistance needed). Item scores were summed to produce a total ADL score ranging from 0 to 22, with higher scores indicating better basic functional capacity. The Cronbach’s alpha coefficient for this scale was 0.898.

Instrumental Activities of Daily Living (IADLs) were assessed using 9 items (B6-1 to B6-9) from the CLASS questionnaire, covering stair climbing, history of falls in the past 12 months, outdoor walking, use of public transportation, shopping, financial management, lifting objects (≥ 5 kg), cooking, and housework. Items were reverse-coded and summed to yield a total IADL score, with higher scores indicating better instrumental functional capacity. The Cronbach’s alpha coefficient for this scale was 0.911.

Cognitive function was assessed using the standardized cognitive assessment indicators available in CLASS 2020. The CLASS survey collected cognitive performance data through a series of standardized cognitive tests covering multiple domains, including orientation, memory, attention, calculation, and recall [34]. Each item was scored on a binary scale (1 = correct response, 0 = incorrect response), and the total cognitive function score was calculated by summing the item scores, ranging from 0 to 16. Higher scores indicate better cognitive functioning. In the present study, this measure demonstrated good internal consistency, with a Cronbach’s alpha coefficient of 0.829.

Following prior research [35], social participation was measured using six items from the CLASS questionnaire assessing the frequency of participation in the following activities over the past year: (a) religious activities; (b) attending senior university or training courses; (c) watching television/listening to radio/reading books or newspapers/listening to opera; (d) singing or playing musical instruments; (e) playing mahjong, chess, or cards; and (f) square dancing. Each item was rated on a five-point frequency scale (0 = never, 1 = a few times a year, 2 = at least once a month, 3 = at least once a week, 4 = almost every day). Items were summed to produce a total social participation score, with higher scores indicating more frequent social engagement. The Cronbach’s alpha coefficient for this scale was 0.842.

Self-efficacy was assessed using 4 items from the CLASS questionnaire, reflecting older adults' perceived capacity to engage with and adapt to social life. Following prior research [36], self-efficacy among older adults is conceptualized as their subjective beliefs and confidence when encountering new things and skills; in the context of aging research using CLASS data, this construct has been operationalized as older adults' confidence in adapting to social change. Items were rated on a five-point Likert scale (1 = completely disagree to 5 = completely agree). The total self-efficacy score ranges from 4 to 20, with higher scores indicating greater self-efficacy. The Cronbach’s alpha coefficient for this scale was 0.762.

### Statistical analysis

R software was used to statistically describe the study population. Continuous variables were represented by mean and standard deviation (SD), and categorical variables were represented by frequency (n) and percentage (%).

Latent profile analysis (LPA) was conducted to identify profiles of digital empowerment among older adults. We used the R package MplusAutomation for LPA [37], with full information maximum likelihood estimators and randomly chosen starting points. As a person-centered method, LPA employs maximum likelihood estimation to assign individuals to groups based on posterior probabilities [38]. This approach provides more precise classifications than traditional clustering techniques. Such accuracy is crucial when studying older adults, whose digital empowerment patterns vary significantly depending on factors like education level, urban or rural residence, and prior exposure to technology.

In order to select the best classification model, the LPA model is usually evaluated with the following metrics: Akaike's information criterion (AIC), Bayesian information criterion (BIC), adjusted Bayesian Information Criterion (aBIC), and entropy. Specifically, smaller AIC, BIC, and aBIC indicate better model fit; Entropy is used to evaluate the classification accuracy of the model. Additionally, all models should exhibit a minimum profile proportion above 5%, a criterion recommended by Tein et al. [39] to ensure representativeness.

To examine the relationship between digital empowerment patterns and SPA, we conducted Ordinary Least Squares (OLS) regression analyses. Statistical analysis was conducted using the R software version 4.1.0.

### Ethical considerations

This study is a secondary analysis based on publicly available data from the China Longitudinal Aging Social Survey (CLASS) [40]. The dataset was accessed for research purposes on January 18, 2025. All personal identifiers were removed by the data provider prior to public release, and the authors had no access to any information that could identify individual participants during or after data collection and analysis. The use of the CLASS dataset complies with the data provider’s ethical requirements and terms of use. The study protocol was reviewed and approved by the Academic Committee of the School of Population and Health, Renmin University of China (Approval ID: L20250106).

## Results

### Results of latent profile analysis

The LPA results indicated that models converged up to five classes, and the six-class model did not converge. As shown in Table 1, the 5-profile model seems to be the most suitable, but one profile the proportional account for few people. Therefore, we considered the 4-class model the optimal one fitting the data. Specially, the 4-class model showed relatively low AIC, BIC, and aBIC scores, as well as the highest entropy scores, which indicated a precise classification accuracy.

**Table 1:** Fit statistics results of the LPA

Number of classes	AIC	BIC	aBIC	Entropy	Class proportion(%)
1	51554.69	51650.96	51600.01	1.000	-
2	45062.48	45212.90	45133.03	0.910	42.64/57.35
3	42946.89	43151.47	43043.11	0.908	23.97/36.67/39.34
4	42183.43	42442.16	42306.21	0.925	18.35/19.52/22.86/39.24
5	41404.57	41717.45	41552.09	0.835	39.22/13.34/23.77/19.11/4.53

### Naming of latent profile

**Fig. 1:**
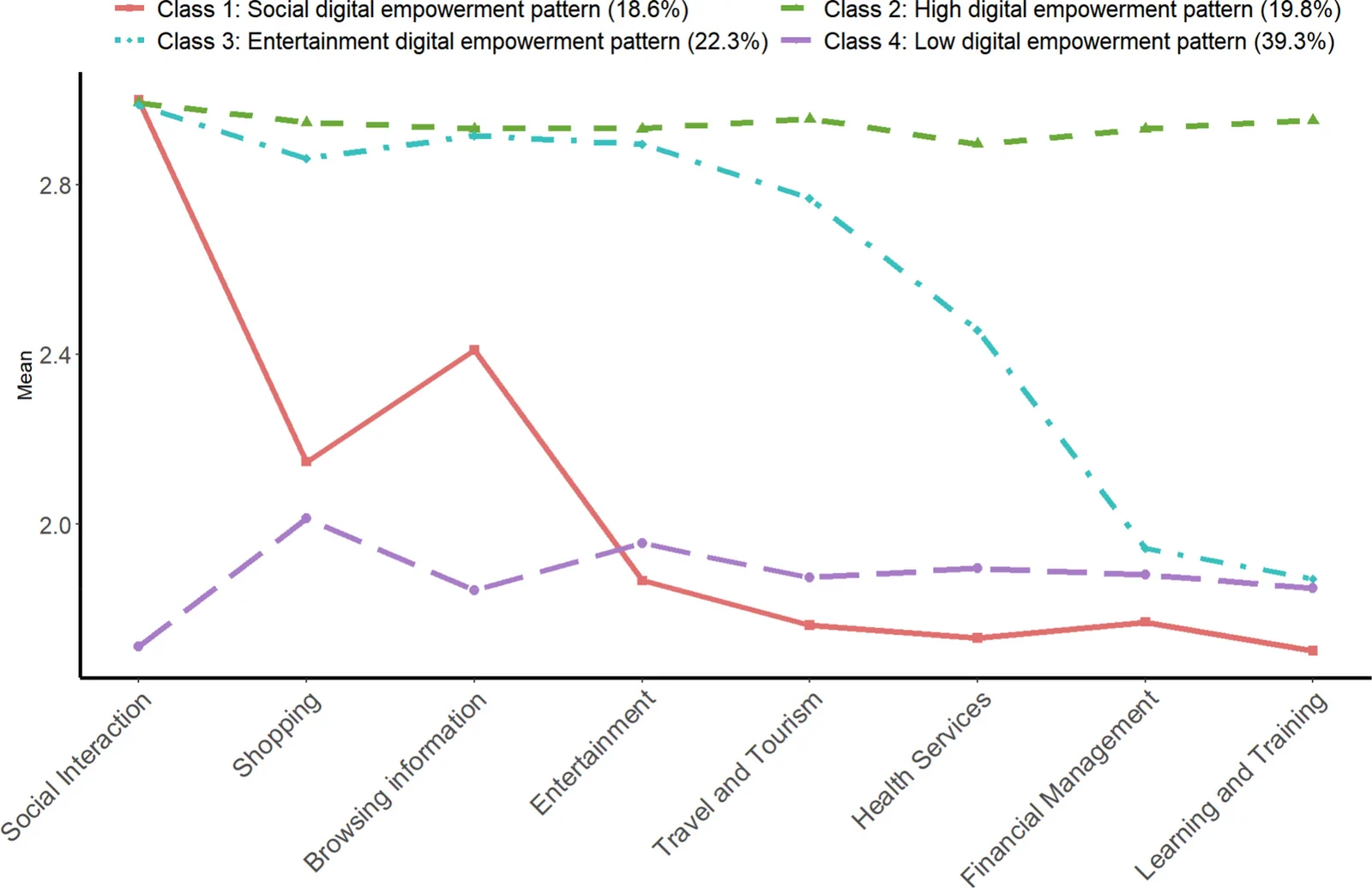
Latent profile model of digital empowerment in older adults

Figure 1 provides the average scores for each item within the 4-profile model of digital empowerment. Class 1, labeled "Social digital empowerment pattern," accounts for 18.6% of the sample and demonstrates high engagement in social interaction but progressively lower engagement across other digital activities, particularly declining in health services and learning and training domains. Class 2, termed the "High digital empowerment pattern," comprises 19.8% of subjects and shows consistently high engagement probabilities across all eight dimensions of digital activities. Class 3, labeled "Entertainment digital empowerment pattern," represents 22.3% of the sample and exhibits high engagement in social interaction, moderate to high engagement in shopping, browsing information, and entertainment, but significantly decreasing engagement in financial management and learning and training. Class 4, designated as the " Low digital empowerment pattern," accounts for the largest proportion at 39.3% and demonstrates consistently low engagement across all digital domains.

### Sample characteristics

Of the 3,032 eligible respondents, the average age was 71.34 ± 6.78 years, with 50.5% female. The SPA mean score was 20.17 (SD = 3.93), and Internet use skills averaged 4.91 (SD = 2.86). 50.5% of the respondents were female, and. More of them were married (76.2%) and lived in the rural (59.3%). 40.7% had primary school education, while only 2.2% had college education or above. Most older adults were not working (78.1%) and lived with others (93.3%). Regarding health conditions, 60.4% had two or more comorbid chronic diseases. The mean ADL score was 21.26 (SD = 2.15) and IADL score was 16.96 (SD = 2.54). The cognitive function mean score was 13.70 (SD = 2.90), and social participation averaged 1.23 (SD = 3.05). Details are provided in Table 2.

**Table 2:** Baseline characteristics of the total sample and the sample by the different profiles^a^

Variables	Overall (*n* = 3032)	Class1 (*n* = 564)	Class2 (*n* = 600)	Class3 (*n* = 677)	Class4 (*n* = 1191)	*p*^b^
SPA, mean (SD)^c^	20.17(3.93)	20.81(3.85)	21.35(3.20)	21.62(3.53)	18.44(3.86)	< 0.001
Internet use skills (SD)^c^	4.91(2.86)	4.50(2.20)	7.29(3.63)	5.65(2.81)	3.47(1.46)	< 0.001
Gender						0.496
Female	1531(50.5)	284(50.4)	287(47.8)	345(51.0)	615(51.6)	
Male	1501(49.5)	280(49.6)	313(52.2)	332(49.0)	576(48.4)	
Age, mean (SD)^c^	71.34(6.78)	72.43(6.93)	68.83(5.79)	70.02(6.35)	72.83(6.89)	< 0.001
Marital status						< 0.001
Other	721(23.8)	164(29.1)	100(16.7)	135(19.9)	322(27.0)	
Married	2311(76.2)	400(70.9)	500(83.3)	542(80.1)	869(73.0)	
Residence						< 0.001
Rural	1235(40.7)	250(44.3)	162(27.0)	241(35.6)	582(48.9)	
Urban	1797(59.3)	314(55.7)	438(73.0)	436(64.4)	609(51.1)	
Educational level						< 0.001
Illiterate	629(20.7)	145(25.7)	63(10.5)	99(14.6)	322(27.0)	
Primary school	1234(40.7)	265(47.0)	174(29.0)	249(36.8)	546(45.8)	
Middle school & High school	1101(36.3)	147(26.1)	335(55.8)	313(46.2)	306(25.7)	
College and above	68(2.2)	7(1.2)	28(4.7)	16(2.4)	17(1.4)	
Family annual income, mean (SD)^c^	9.10(0.83)	9.20(0.67)	9.30(0.75)	8.93(0.83)	9.04(0.92)	< 0.001
Current work status						< 0.001
Not working	2367(78.1)	398(70.6)	464(77.3)	536(79.2)	969(81.4)	
Working	665(21.9)	166(29.4)	136(22.7)	141(20.8)	222(18.6)	
Living alone						< 0.001
None	2829(93.3)	516(91.5)	580(96.7)	640(94.5)	1093(91.8)	
Yes	203(6.7)	48(8.5)	20(3.3)	37(5.5)	98(8.2)	
ADLs, mean (SD)^c^	21.26(2.15)	21.58(1.58)	21.32(2.18)	21.43(1.74)	20.99(2.51)	< 0.001
IADLs, mean (SD)^c^	16.96(2.54)	17.13(2.33)	17.36(2.12)	17.33(2.19)	16.48(2.92)	< 0.001
Number of comorbid chronic disease						< 0.001
0	392(12.9)	54(9.6)	83(13.8)	88(13.0)	167(14.0)	
1	809(26.7)	185(32.8)	178(29.7)	150(22.2)	296(24.9)	
≥ 2	1831(60.4)	325(57.6)	339(56.5)	439(64.8)	728(61.1)	
Cognitive function, mean (SD)^c^	13.70(2.90)	13.60(2.85)	14.82(2.27)	14.27(2.60)	12.85(3.11)	< 0.001
Social participation, mean (SD)^c^	1.23(3.05)	1.01(2.92)	1.65(3.43)	1.65(3.29)	0.88(2.70)	< 0.001
Self-efficacy (SD)^c^	11.87(3.21)	12.38(3.15)	12.96(3.25)	11.57(3.52)	11.26(2.85)	< 0.001
Living region						
Eastern	2041(67.3)	360(63.8)	441(73.5)	416(61.4)	824(69.2)	< 0.001
Central	733(24.2)	141(25.0)	111(18.5)	211(31.2)	270(22.7)	< 0.001
Western	258(8.5)	63(11.2)	48(8.0)	50(7.4)	97(8.1)	0.085

The definition of the classes: Class 1: Social digital empowerment pattern; Class 2: High digital empowerment pattern; Class 3: Entertainment digital empowerment pattern; Class 4: Low digital empowerment pattern

*SPA* self-perception of aging

^a^Data are presented as counts (percentage) unless otherwise indicated

^b^*P* value determined using χ2 test or analysis of variance F-test

^c^For continuous variables, mean (SD) for each group and significance from analysis of variance F-test are reported

Based on the results of LPA, the sample was categorized into four distinct digital empowerment profiles: Class 1 (Social digital empowerment pattern) comprising 564 participants (18.6%), Class 2 (High digital empowerment pattern) including 600 participants (19.8%), Class 3 (Entertainment digital empowerment pattern) containing 677 participants (22.3%), and Class 4 (Low digital empowerment pattern) representing 1,191 participants (39.3%).

Class 4 (Low digital empowerment pattern) exhibited the lowest SPA score (18.44) along with other distinctive characteristics that suggested significant barriers to digital technology adoption, including the lowest Internet use skills score (3.47), oldest mean age (72.83 years), highest proportion of illiteracy (27.0%), and highest proportion of rural residence (48.9%). This group also demonstrated challenges in functional abilities, with the lowest ADLs (20.99) and IADLs (16.48) scores, and limited cognitive function (12.85), social participation (0.88), and self-efficacy (11.26). In contrast, Class 2 (High digital empowerment) represented the most technologically engaged segment, characterized by the highest Internet use skills score (7.29), youngest mean age (68.83 years), highest educational level (with 55.8% having middle school education and 4.7% having college education), and greatest urban representation (73.0%). This group also exhibited better functional status, with the highest IADLs (17.36) scores, highest cognitive function score (14.82), and highest self-efficacy (12.96).

Notably, significant differences were observed across other sociodemographic characteristics and health indicators, including age, marital status residence, educational level, family annual income, current work status, living alone status, physical functioning measured by ADLs and IADLs, number of comorbid chronic diseases, cognitive function, social participation, and self-efficacy, as well as living region (Eastern and Central regions).

### Relationship between digital empowerment patterns and SPA

Table 3 presents the OLS regression results examining the relationship between digital empowerment patterns and SPA, using Class 4 (Low digital empowerment pattern) as the reference group. Model 1 was adjusted only for the digital empowerment pattern variable. Model 2 further adjusted for sociodemographic and health-related characteristics that may be associated with SPA among older adults, including gender, age, marital status, place of residence, education level, annual household income, employment status, living arrangement, ADLs, IADLs, number of chronic diseases, cognitive ability, social participation, and self-efficacy. Model 3 additionally considered residential region to examine whether regional differences affected the observed associations.

**Table 3:** OLS regression of digital empowerment patterns on SPA (Reference: Class 4: Low digital empowerment pattern)

Variable	Model 1	Model 2	Model 3
Class1	2.367^***^ (0.187)	1.772^***^ (0.178)	1.771^***^ (0.172)
Class2	2.907^***^ (0.183)	2.021^***^ (0.189)	2.077^***^ (0.182)
Class3	3.182^***^ (0.176)	2.599^***^ (0.171)	2.431^***^ (0.165)
Gender (Ref. = Female)		−0.005 (0.128)	0.074 (0.123)
Age		0.013 (0.012)	−0.003 (0.011)
Marital status (Ref. = Others)		−0.224 (0.183)	−0.180 (0.177)
Residence (Ref. = Rural)		−0.503^***^ (0.156)	−0.088 (0.153)
Education (Ref. = Illiterate)			
Primary school		0.003 (0.173)	−0.373^**^ (0.169)
Middle school & High school		−0.077 (0.203)	−0.246 (0.196)
College and above		1.335^***^ (0.455)	1.241^***^ (0.439)
Family annual income		0.045 (0.078)	0.217^***^ (0.076)
Employment (Ref. = unemployment)		0.639^***^ (0.178)	0.129 (0.175)
Living alone (Ref. = No)		−0.371 (0.292)	−0.572^**^ (0.282)
ADLs		0.125^***^ (0.048)	0.112^**^ (0.047)
IADLs		0.170^***^ (0.044)	0.136^***^ (0.042)
Number of comorbid chronic disease		0.155^*^ (0.090)	0.007 (0.088)
Cognitive ability		0.286^***^ (0.026)	0.267^***^ (0.025)
Social participation		0.049^**^ (0.021)	0.041^**^ (0.021)
Self-efficacy		0.123^***^ (0.021)	0.069^***^ (0.021)
Living region (Ref. = Western)			
East			−2.047^***^ (0.232)
Central			0.260 (0.244)
N	3032	3032	3032
R^2^	0.129	0.247	0.302

The definition of the classes: Class 1: Social digital empowerment pattern; Class 2: High digital empowerment pattern; Class 3: Entertainment digital empowerment pattern; Class 4: Low digital empowerment pattern

^*^p < 0.05, **p < 0.01, ***p < 0.001

The analysis showed that digital empowerment patterns were significantly and positively associated with SPA among older adults, and this association remained robust across all three models. Class 1, Class 2, and Class 3 were positively correlated with SPA relative to Class 4.

In Model 3, education level (with college education showing a strong positive effect of 1.241, *p* < 0.001), family annual income (0.217, *p* < 0.001), ADLs (0.112, *p* < 0.01), IADLs (0.136, *p* < 0.001), cognitive ability (0.267, *p* < 0.001), social participation (0.041, *p* < 0.01), and self-efficacy (0.069, *p* < 0.001) showed strong positive associations with SPA, while living alone (− 0.572, *p* < 0.01) and eastern region (− 2.047, *p* < 0.001) showed strong negative associations.

### Heterogeneity analysis

Table 4 presents a heterogeneity analysis examining how various digital empowerment patterns influence SPA across different demographic subgroups, using Class 4 (Low digital empowerment pattern) as the reference group.

**Table 4:** Heterogeneity analysis (Reference: Class 4: Low digital empowerment pattern)

	Sex		Residence		Education		
	Female	Male	Rural	Urban	Illiterate	Primary school	Middle school and above
Class 1	1.674*** (0.239)	1.852*** (0.249)	1.638*** (0.260)	1.975*** (0.232)	2.297*** (0.356)	1.575*** (0.247)	1.616*** (0.323)
Class 2	1.990*** (0.257)	2.163*** (0.260)	2.463*** (0.317)	2.210*** (0.232)	2.592*** (0.501)	2.000*** (0.293)	1.732*** (0.279)
Class 3	2.848*** (0.228)	1.999*** (0.241)	2.722*** (0.267)	2.319*** (0.215)	3.665*** (0.408)	1.914*** (0.252)	2.199*** (0.266)
N	1531	1501	1235	1797	629	1234	1169
R^2^	0.321	0.275	0.336	0.237	0.407	0.305	0.238

OLS estimates are reported; All control variables are controlled

The definition of the classes: Class 1: Social digital empowerment pattern; Class 2: High digital empowerment pattern; Class 3: Entertainment digital empowerment pattern; Class 4: Low digital empowerment pattern

^***^p < 0.001

For sex-based differences, both females and males demonstrate more positive SPA across all three digital empowerment patterns compared to the Class 4. Notably, males show stronger positive effects in Class 1 (Social digital empowerment pattern) and Class 2 (High digital empowerment pattern) compared to females. However, females experience the strongest positive effect from Class 3 (Entertainment digital empowerment pattern). This suggests that while males benefit more from social and high digital empowerment patterns, entertainment-oriented digital empowerment may be particularly beneficial for women's attitudes toward aging.

Regarding residence-based differences, both rural and urban residents have a profound impact on SPA across all digital empowerment patterns. Rural older adults exhibit particularly pronounced benefits from Class 2 and Class 3 compared to their urban counterparts. These patterns suggest that geographical context substantially moderates how digital empowerment influences attitudes toward aging.

Education level analysis reveals the most pronounced differences, with illiterate individuals showing dramatically higher coefficients across all patterns compared to those with primary education or middle school and above.

### The moderating effect of Internet use skills

Table 5 reveals the moderation effect analysis of Internet use skills on digital empowerment patterns and SPA. All three digital empowerment patterns show significant positive associations with SPA compared to the Class 4.

**Table 5:** The results of the moderation effect analysis (*N* = 3032)

Variables	Model 1	Variables	Model 1
Class 1	1.184 **(0.410)	Interaction 1	0.174(0.092)
Class 2	1.185**(0.403)	Interaction 2	0.199**(0.077)
Class 3	1.679***(0.391)	Interaction 3	0.187*(0.081)
Internet use skills	−0.003(0.023)	${R}^{2}$	0.381

All control variables are controlled. The definition of the classes: Class 1: Social digital empowerment pattern; Class 2: High digital empowerment pattern; Class 3: Entertainment digital empowerment pattern; Class 4: Low digital empowerment pattern

^*^p < 0.05, **p < 0.01, ***p < 0.001

Class 2 (High digital empowerment pattern) shows the strongest moderation effect, with a highly significant interaction term (0.199, *p* < 0.01), indicating that Internet use skills strongly influence how high digital empowerment affects SPA. The interaction with Class 3(0.187, *p* < 0.05) is significant, while the interaction with Class 1 is not statistically significant.

**Fig. 2:**
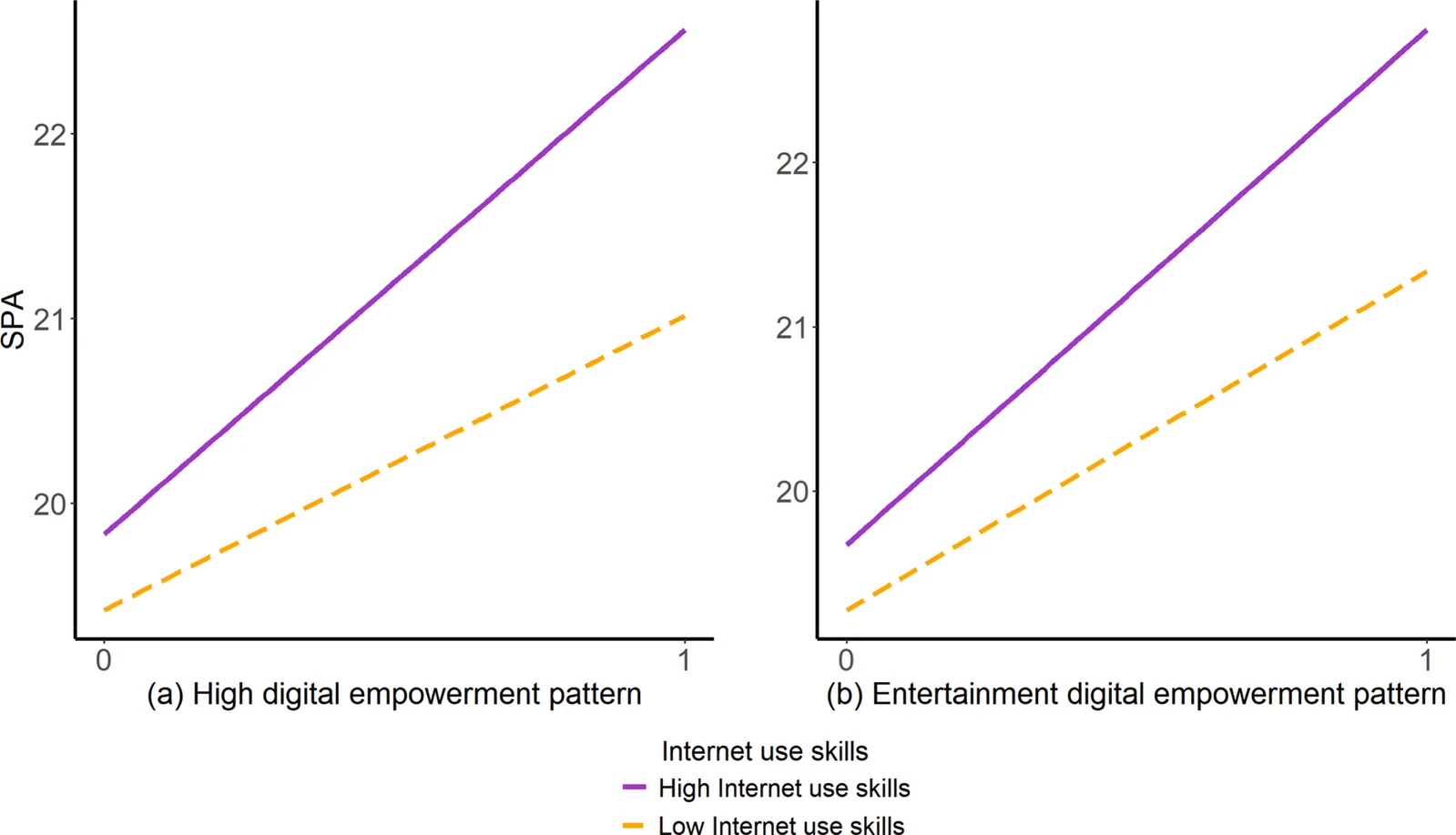
Internet use skills moderate the relation between digital empowerment patterns and SPA. Note. SPA = self-perception of aging

Figure 2 visually confirms this relationship, showing divergent patterns between high and low Internet use skills groups. Individuals with high Internet skill (solid lines) demonstrates substantially more positive SPA when engaged in digital empowerment patterns, while those with low Internet use skills (dashed lines) show more modest improvements in SPA. This is particularly evident in Class 2 and Class 3, with the gap between high and low Internet use skills groups widening as digital engagement level increases (panel a and panel b).

## Discussion

This study investigated the relationship between digital empowerment patterns and SPA among older Chinese adults using latent profile analysis. Further examination revealed distinct characteristics across four digital empowerment profiles: Class 1 (Social digital empowerment pattern, 18.6%), Class 2 (High digital empowerment pattern, 19.8%), Class 3 (Entertainment digital empowerment pattern, 22.3%), and Class 4 (Low digital empowerment pattern, 39.3%). The high digital empowerment pattern (Class 2) demonstrated the highest Internet use skills score, the youngest mean age, highest proportion of married individuals, urban residents, highest educational attainment, the highest IADLs score, highest cognitive function score, and highest self-efficacy. In contrast, Class 4 (Low digital empowerment pattern), representing the largest segment, exhibited the lowest SPA score, the lowest Internet use skills score, the oldest mean age, the highest proportion of illiteracy, rural residence, the lowest ADLs and IADLs scores, limited cognitive function, social participation, and self-efficacy. The mean SPA score for Class 3 (M = 21.62, SD = 3.53) exceeded that of Class 4 (M = 18.44, SD = 3.86) by 3.18 points.

Our findings reveal that patterns of different digital empowerment patterns are significantly associated with SPA. Among these, Class 3 (entertainment digital empowerment pattern) shows the strongest positive association with SPA, followed by Class 2 and Class 1. This aligns with the findings of Mariano et al. [41], who found that greater use of information and communication technology to be generally associated with positive SPA. The Internet provides older adults with opportunities to maintain contact with family and friends and access social support, which may help reduce loneliness and foster more positive perceptions of aging [42]. Furthermore, online platforms strengthen social capital by expanding the social networks among older adults, thereby contributing to more positive self-assessments of aging [43].

Notably, the heterogeneity analysis revealed important demographic variations in the relationship between digital empowerment patterns and SPA. Compared with women, men appeared to derive greater benefits from Class 1 (social digital empowerment pattern) and Class 2 (high digital empowerment pattern), whereas the association was strongest between Class 3 (entertainment digital empowerment pattern) and SPA among women. The previous study by Wang and Gu [44], who reported that the positive association between social media use and SPA was significant only among older men. Such gender difference could be attributed to older men tend to use digital technologies for information seeking, financial management, and social network expansion [45]. Such patterns may increase the benefits derived from socially and informationally oriented forms of digital empowerment by facilitating social participation and access to resources. In contrast, older women may be more likely to engage in entertainment-oriented and emotionally rewarding online activities, which can contribute to emotional well-being and leisure satisfaction. Consequently, entertainment-oriented digital engagement may play a more prominent role in promoting SPA among older women.

Geographically, rural older adults showed consistently stronger associations between digital empowerment patterns and positive SPA compared to their urban counterparts, particularly for Class 2 (high digital empowerment pattern) and Class 3 (entertainment digital empowerment pattern). One possible explanation is that opportunities for offline social participation and access to services are often more limited in rural areas, making digital technologies a more important resource for everyday life. Consequently, higher levels of digital empowerment may provide greater benefits for SPA among rural older adults. This finding is in line with Xiang et al. [46], who reported a stronger positive association between smartphone use and aging attitudes among rural than urban older adults.

Educational differences were also observed. Compared with those with higher levels of education, older adults with lower educational attainment showed stronger associations between digital empowerment patterns and SPA. One possible explanation is that individuals with less formal education have traditionally faced greater barriers to accessing information, social resources, and opportunities for participation. Digital technologies may help reduce some of these barriers by providing new channels for information acquisition, social interaction, and engagement in cultural and community activities. As a result, the benefits associated with digital empowerment may be more pronounced among older adults with lower educational attainment.

In addition, Internet use skills played a significant moderating role in the association between digital empowerment patterns and SPA, particularly for the Class 2 (high digital empowerment pattern) and Class 3 (entertainment digital empowerment pattern). One possible explanation is that older adults with higher Internet use skills often have higher self-efficacy, which contributes to greater adaptability and more positive views on aging.

Several limitations of this study should be noted. First, given the cross-sectional nature of the data, this study was unable to capture changes in the relationship between digital empowerment and SPA over time. Therefore, the findings should be interpreted with caution, as the study design limits causal inference and leaves open the possibility of reverse causality or residual confounding. Future research would benefit from using longitudinal data with multiple waves of follow-up to examine temporal changes, clarify the direction of associations, and provide stronger evidence regarding the potential causal relationship between digital empowerment and SPA. Second, the use of self-reported questionnaires may introduce response bias, including recall bias and social desirability bias, which could affect the accuracy of the measured variables. In addition, unmeasured factors such as digital self-efficacy and general health status may also confound the observed associations between digital empowerment and SPA, and should be considered in future studies. Third, given the constraints of the available secondary data, the measurement of digital skills is based on only three device-related items (smartphone, tablet, and computer), which may not fully capture the multidimensional nature of functional digital literacy among older adults.

Despite these limitations, the present study has several notable strengths. To our knowledge, this is the first personal-centered analysis of digital empowerment patterns and SPA among older adults in China, which can provide a more nuanced understanding of individual differences and contribute to personalized intervention and treatment strategies. A further strength is the use of large-scale, nationally representative CLASS data, which enhances the generalizability of the findings.

Based on these findings, several policy implications can be proposed to reduce digital inequality among older adults and promote more positive perceptions of aging through digital engagement. First, improving the age-friendliness of digital products, particularly applications related to entertainment, social interaction, and learning, with intuitive interfaces, larger text sizes, simplified navigation, and clear instructions to accommodate the needs of older users. Second, digital engagement strategies should consider demographic heterogeneity among older adults. For example, entertainment-oriented digital activities may be particularly beneficial for older women, whereas broader forms of digital empowerment involving social, informational, and practical functions may be more relevant for older men. For rural older adults, expanding access to both high-level digital services and entertainment-related platforms may help compensate for limited offline resources and support social participation. Third, given the moderating role of Internet use skills identified in this study, efforts should be directed toward improving digital literacy among older adults. Community-based training programs, intergenerational learning initiatives, and targeted public interventions may help enhance digital skills, especially among older adults with lower educational attainment, who may experience greater benefits from digital empowerment.

## Conclusion

This study examined the relationship between digital empowerment patterns and SPA among older adults in China. Using latent profile analysis, we identified distinct digital empowerment profiles and found that higher levels of digital empowerment were positively associated with SPA.

These findings highlight the need for tailored digital inclusion policies for older adults. Interventions for older women may focus on emotional support and digital confidence, while community-based programs with offline assistance may better serve rural and less-educated older populations. Promoting age-friendly digital design may help reduce usability barriers.

## Data Availability

No datasets were generated or analysed during the current study.
